# Association of intestinal colonization of ESBL-producing *Enterobacteriaceae* in poultry slaughterhouse workers with occupational exposure—A German pilot study

**DOI:** 10.1371/journal.pone.0232326

**Published:** 2020-06-04

**Authors:** Katharina Wadepohl, Anja Müller, Diana Seinige, Karl Rohn, Thomas Blaha, Diana Meemken, Corinna Kehrenberg

**Affiliations:** 1 Field Station for Epidemiology, University of Veterinary Medicine Hannover, Bakum, Germany; 2 Institute for Food Quality and Food Safety, University of Veterinary Medicine Hannover, Hannover, Germany; 3 Institute for Biometry, Epidemiology and Information Processing (IBEI), University of Veterinary Medicine Hannover, Foundation, Hannover, Germany; 4 Institute of Food Safety and Food Hygiene, Section Meat Hygiene, Department of Veterinary Medicine, Freie Universität Berlin, Berlin, Germany; 5 Institute for Veterinary Food Science, Faculty of Veterinary Medicine, Justus-Liebig-University, Giessen, Germany; University Hospital Basel, SWITZERLAND

## Abstract

**Background:**

Bacteria that have acquired antimicrobial resistance, in particular ESBL-producing *Enterobacteriaceae*, are an important healthcare concern. Therefore, transmission routes and risk factors are of interest, especially for the carriage of ESBL-producing *E*. *coli*. Since there is an enhanced risk for pig slaughterhouse employees to carry ESBL-producing *Enterobacteriaceae*, associated with animal contact as potential risk factor, the present study investigated the occurrence of ESBL-producing *Enterobacteriaceae* in poultry slaughterhouse employees. Due to the higher level of resistant *Enterobacteriaceae* in primary poultry production than in pig production, a higher risk of intestinal colonization of poultry slaughterhouse employees was expected.

**Results:**

ESBL-producing *Enterobacteriaceae* were detected in 5.1% (5 of 99) of the fecal samples of slaughterhouse workers. The species of these isolates was confirmed as *E*. *coli*. PCR assays revealed the presence of the genes *bla*_CTX-M-15_ (n = 2) and *bla*_SHV-12_ (n = 3) in these isolates, partly in combination with the β-lactamase gene *bla*_TEM-135_. Participants were divided into two groups according to their occupational exposure and results indicated an increased probability of colonization with ESBL-producing *Enterobacteriaceae* for the group of ‘higher exposure’ (OR 3.7, exact 95% CI 0.6–23.5; p = 0.4). For intestinal colonization with ESBL-producing *Enterobacteriaceae*, a prevalence of 10% (3/30) was observed in the group of ‘higher exposure’ versus 2.9% (2/69) in the group of ‘lower exposure’. Employees in working steps such as ‘hanging’ poultry in the process of slaughter and ‘evisceration’ seemed to have a higher risk for intestinal colonization with ESBL-producing *Enterobacteriaceae* compared to the group of ‘lower exposure’.

**Conclusion:**

This study is the first of its kind to collect data on the occupational exposure of slaughterhouse workers to ESBL-producing *Enterobacteriaceae* in Europe. The results suggested that colonization with ESBL-producing *Enterobacteriaceae* is associated with occupational exposure in poultry slaughterhouses. However, the presence of ESBL-producing *E*. *coli* isolates in only 5.1% (5/99) of the tested employees in poultry slaughterhouses suggests a lower transmission risk than in pig slaughterhouses.

## Introduction

Antimicrobial resistant bacteria in animals are a challenge for public health, as they can be transferred from animals to humans via direct contact or indirectly through the food chain [1, 2]. In addition, mobile resistance elements can be transferred between bacteria and might contribute to the spread of bacterial resistance. In humans, infections with extended-spectrum β-lactamase (ESBL-)producing *Enterobacteriaceae* in particular are associated with an increased burden of disease [3]. Since the 1990s, there has been a shift in ESBL-genes associated with nosocomial and health-related infections from genes mainly of TEM and SHV groups to genes of the CTX-M group. [4]. The ESBL gene *bla*_CTX-M-15_ is currently the most common ESBL gene type in human *E*. *coli*, although the number of carriers of *bla*_CTX-M-1_ genes have continued to increase in recent years [5]. Differences were found in the frequency of detection of certain ESBL genes in *E*. *coli* from humans and animals. While *bla*_CTX-M-15_ is the most common ESBL gene found in clinical *E*. *coli* isolates from humans, the *bla*_CTX-M-1_ gene is the most frequent gene among *E*. *coli* from livestock [6, 7]. The ESBL-gene *bla*_SHV_ and AmpC β-lactamase gene *bla*_CMY-2_ are found more frequently in isolates from poultry than in isolates from other livestock [8]. Furthermore, the level of ESBL-producing *E*. *coli* seems to be higher in poultry flocks than in other livestock such as pigs and cattle [6, 7, 9, 10].

The presence of ESBL-producing *Enterobacteriaceae* in animals is a potential risk for humans that are occupationally exposed. In the case of poultry farming, this mainly concerns the farmers and slaughterhouse workers [11–13]. A study by Dohmen et al. (2017) showed that the risk of intestinal colonization with ESBL-producing *Enterobacteriaceae* of slaughterhouse workers in pig slaughterhouses depends on their tasks, in particular whether they have close and frequent contact to live animals, carcasses or organs. Based on these findings and considering the high prevalence of ESBL-producing *Enterobacteriaceae* in poultry flocks, the risk of intestinal colonization with ESBL-producing *Enterobacteriaceae* in workers employed in poultry slaughterhouses might be even higher than in other livestock sectors. Price et al. (2007) [14] showed that the occupational exposure might be a possible route of transmission as these authors were able to demonstrate increased rates of colonization with gentamicin-resistant *E*. *coli* in U.S. poultry workers compared with community references. However, no previous study investigated the occurrence of ESBL-producing *E*. *coli* as intestinal colonizer in the context of occupational exposure of poultry slaughterhouse workers in Europe. Therefore, we investigated the occurrence of ESBL-producing *E*. *coli* as an intestinal colonizer in poultry slaughterhouse workers, working at different stages of the slaughter process.

## Materials and methods

### Study design, bacterial isolation and ESBL confirmation

As a pilot study, a medium-sized broiler slaughterhouse (250 employees/120000 chickens/day) in the northeast of Germany, slaughtering only poultry fattened in Germany, was visited by our research team in November 2017. All employees of the slaughterhouse involved in the slaughter process (n = 99) were asked to participate in the study. The participants had to answer a questionnaire and to hand in a stool sample. The questionnaire included questions on personal and occupational data, e.g., working position in the slaughterhouse. Due to the different nationalities of the workers at the slaughterhouse, the questionnaire was translated into Polish, Russian and Rumanian to ensure that the questions asked were fully understood. Before submitting their stool specimen and questionnaire, all participants had given written declaration of consent, that was then officially approved by the Medical Ethical Committee of Lower Saxony, Germany (document no. BO/32/2014). All procedures contributing to this study complied with the Declaration of Helsinki (1975), as revised in 2008, as well as with relevant national ethical standards.

Stool samples were taken on the day of the visit by the research team and kept cooled at 4 °C from collection until arrival in the laboratory on the same day. The samples were tested using selective plating, following the protocol by Dohmen et al. (2017) with the modification of using ESBL Brilliance^®^ agar plates (Oxoid, Wesel, Germany) instead of MacConkey agar plates with cefotaxime (1 μg/mL). From each agar plate, all colonies with different morphology were included in the study. Presumptive colonies were tested for ESBL-production by using a phenotypic confirmatory test for ESBL-producing *Enterobacteriaceae* as recommended by the Clinical and Laboratory Standards Institute (CLSI) [15]. The disk diffusion test was performed measuring the zones of inhibition surrounding 30 μg cefotaxime (CTX) and 30 μg ceftazidime (CAZ) disks with or without clavulanate (CA). An increase of ≥5 mm in zone diameters for cefotaxime and ceftazidime with clavulanate compared to the results when the antimicrobials were tested alone indicated ESBL-production. After confirmation of phenotypic ESBL-production, the species of all isolates was determined by using MALDI-TOF mass spectrometry (Bruker Daltonics, Bremen, Germany). The presence of ESBL-genes was investigated by using previously described PCR assays, targeting the β-lactamase encoding genes *bla*_TEM_, *bla*_CMY-2_, *bla*_OXA-2_, *bla*_SHV_ [16] and *bla*_CTX-M_ [17]. All isolates that showed positive results with the universal CTX-M primers were further tested with group- specific primers for CTX-M groups 1, 2, 8, 9 and 25 [18, 19]. In addition, the presence of AmpC-β-lactamase genes from different groups (ACC, CIT, DHA, EBC, FOX, MOX) as well as OXA-1-like β-lactamase genes was investigated using primers published by Dallenne et al. (2010) [20]. The amplicons were sequenced on both strands (Eurofins Genomics, Ebersberg, Germany) in order to determine the specific β-lactamase genes present in each isolate.

Sequences were compared to sequences in the database using BLAST (https://blast.ncbi.nlm.nih.gov/).

Afterwards, the genetic relatedness of all ESBL-producing *E*. *coli* isolates was tested using pulsed-field gel electrophoresis (PFGE) in accordance with the PulseNet protocol for *Escherichia coli* O157:H7, *Salmonella*, and *Shigella* [21]. Genomic DNA was digested with XbaI. Band patterns were analyzed using the Bionumerics software version 7.6 (Applied Maths, Sint-Martens-Latem, Belgium), applying the Dice coefficient with 0.5%, optimization and 1% position tolerance.

### Statistical analysis

All slaughterhouse workers carrying an ESBL-producing *E*. *coli* isolate with a confirmed ESBL-gene were classified as ESBL positive. The statistical evaluation was carried out using SAS 9.4m5 with the Enterprise Guide Client 7.15 (SAS Institute Inc., Cary, NC, USA).

To improve the comparability of studies, the analysis was based on the conceptual framework of the statistical analysis proposed by Dohmen et al. (2017). Therefore, slaughterhouse workers were divided into two groups according to the potential exposure during the performance of their main task in the slaughter process. The group with potentially higher exposure to ESBL-producing *Enterobacteriaceae* consisted of job tasks with direct contact to poultry or carcasses (e.g., hanging, bleeding, evisceration). The group with an assumed lower exposure included all tasks after cooling such as packaging and expedition. For participants who had reported having more than one task in the slaughter process, the task with the highest exposure was considered for the categorization.

For dichotomous variables, univariable analysis was performed using Chi-Square test. For potential risk or confounding factors, such as nationality, consumption of raw meat, hospitalization, recent use of antimicrobials, living on a farm, traveling, gender and smoking habits were analyzed univariate by calculating odds ratios of intestinal colonization with ESBL-producing *E*. *coli* in slaughterhouse workers in different working positions by means of cross tables. ‘Age’, as a metric parameter was analysed using a t-test.

Missing values in the tables results from incomplete answers in the questionnaire, therefore the number (n) for each characteristic varied, as shown in Fig 2. For characteristics (use of antimicrobials and a hospitalization in the past 12 months), which did not apply for any of the employees colonized with ESBL-producing *Enterobacteriaceae* the odds ratio has not been calculated (N/A). Differences were significant at p ≤ 0.05.

## Results

**Fig 1:**
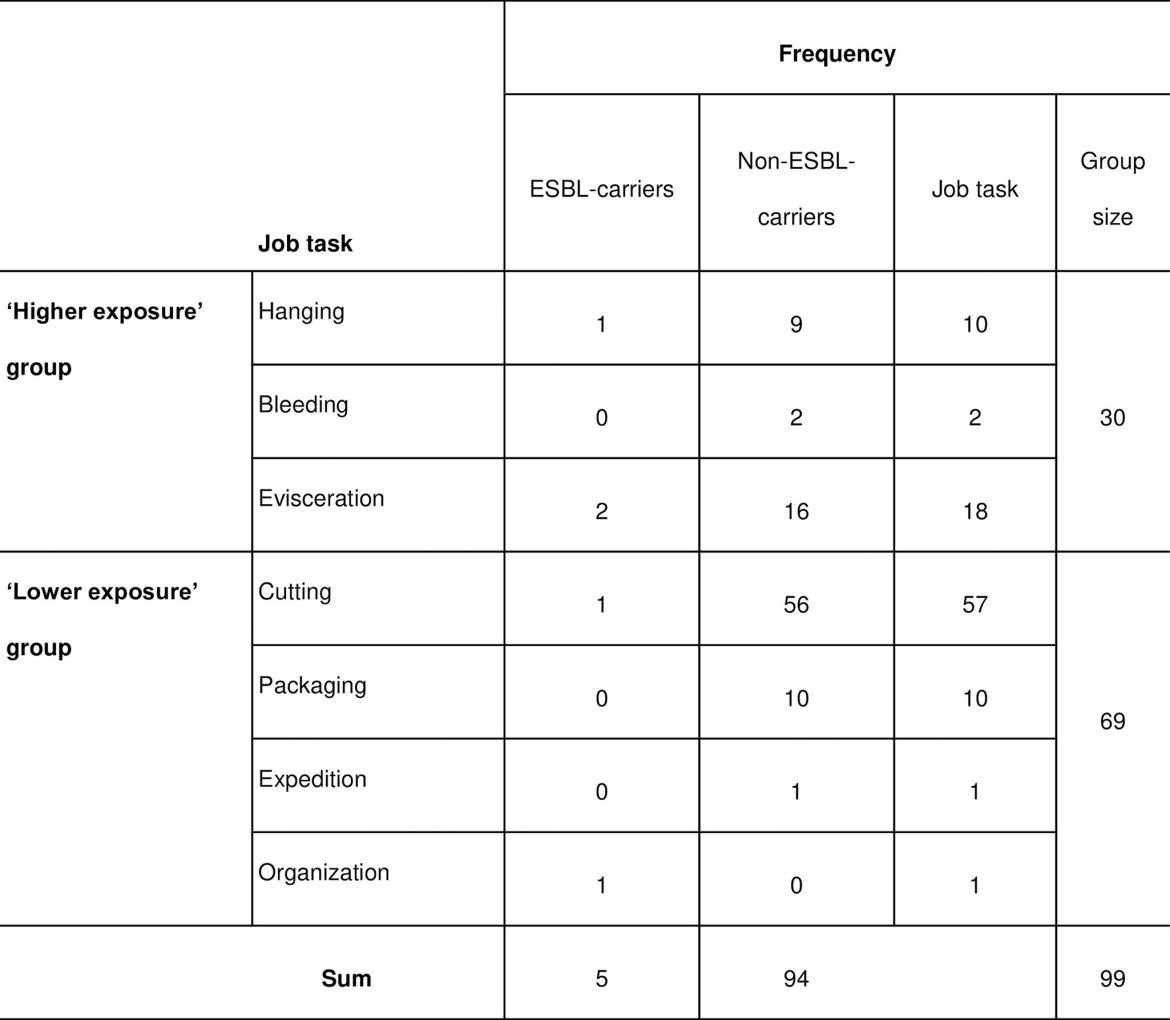
Exposure groups and ESBL carriage of slaughterhouse workers with different job tasks. Data presented as number of workers divided into two groups by their status of intestinal colonization with extended-spectrum β-lactamase (ESBL)-producing *E*. *coli*.

A total of 99 slaughterhouse employees were recruited to participate in the study. The number of participants (n = 30) of the group of high exposure was only one third of the overall number of participants (cf. Fig 1).

**Fig 2:**
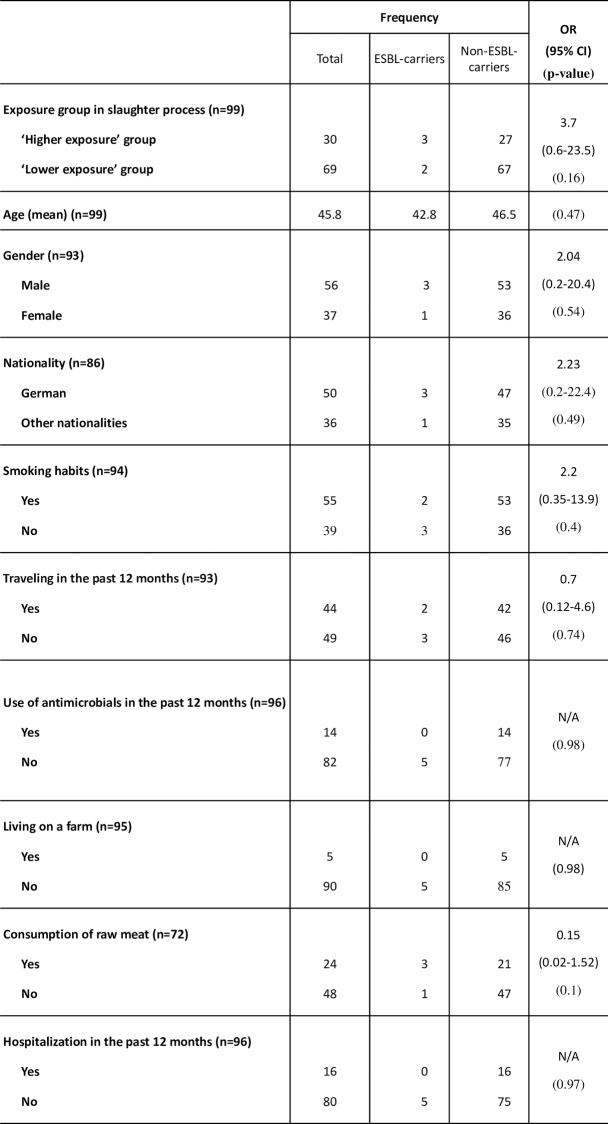
Characteristics of participants and ESBL carriage. Results of univariate analysis. Data presented as number of workers divided into two groups by their status of intestinal colonization with extended-spectrum β-lactamase (ESBL)-producing *E*. *coli* and further work and personal related characteristics; OR—odds ratio; CI—confidence interval. ^1^Not applicable (N/A), used for characteristics for which odds ratios could not be calculated due to one column being zero.

The origin of the participants was nearly equally divided between German and other nationalities (Fig 2). The most frequent participating nationality apart from German (n = 50) was Polish (n = 34), followed by employees of Belarusian (n = 1), Armenian (n = 1) and Guinean (n = 1) nationality participated. In total, 60% of participants were male and 40% female.

**Fig 3:**
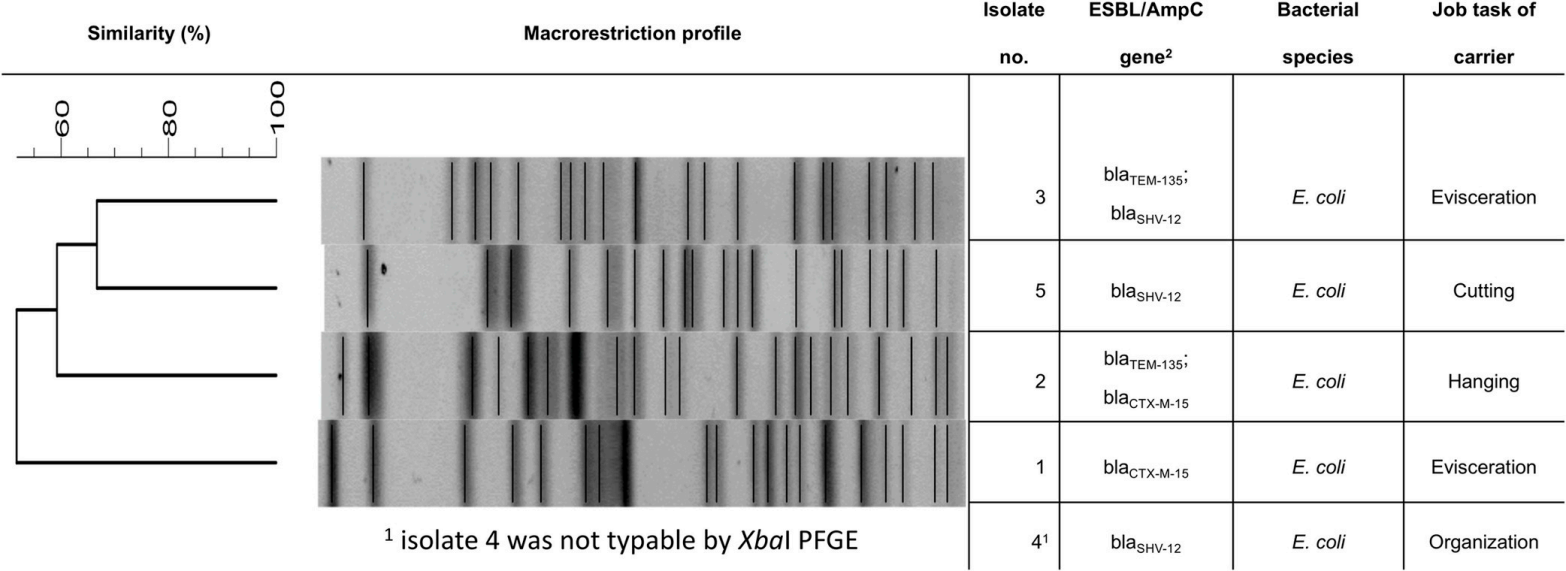
Characteristics of the ESBL-producing *E*. *coli* isolates (isolate numbers correspond to participant numbers). ^1^ isolate 4 was not typable by *Xba*I PFGE; ^2^ the TEM-135 β-lactamase lacks ESBL activity.

From all 99 participants, fecal samples were analyzed. Presumptive ESBL-producing bacteria were collected on selective agar plates from five of the stool samples (5.1%). The species determination by MALDI-TOF analysis identified these isolates as *E*. *coli* and the production of ESBL enzymes was verified by the disk diffusion phenotypic confirmatory test. The β-lactamase genes detected by PCR assays and sequencing were *bla*_CTX-M-15_ (n = 2), *bla*_TEM-135_ (n = 2) and *bla*_SHV-12_ (n = 3). Two of the isolates (from participants 2 and 3) carried more than one β-lactamase gene. However, the additional genes detected in the two isolates were *bla*_TEM-135_ genes, which lack ESBL activity and only code for a β-lactamase with narrow spectrum of activity [22]. However, both isolates carried an ESBL type β-lactamase in combination with *bla*_TEM-135_ (*bla*_TEM-135_ and *bla*_CTX-M-15_ or *bla*_TEM-135_ and *bla*_SHV-12_, respectively). The PFGE band patterns showed a similarity of 60% between the isolates of participants 2, 3 and 5 and approximately 67% between isolates 3 and 5 (Fig 3) and greater than 50% similarity with isolate 1. One isolate, obtained from worker 4, repeatedly did not yield analyzable band patterns and was regarded as non-typable by *Xba*I macrorestriction analysis.

The analysis of a possible association between the occupational exposure of study participants and ESBL carriage seems to indicate, that the characteristics smoking habit, nationality and traveling could have an influence, as shown in Fig 2. Other characteristics of the questionnaire, such as use of antimicrobials and hospitalization within in the last 12 months and living on a farm and consumption of raw meat, could either not be interpreted due to zero findings or showed no association with the carriage of ESBL-producing *E*. *coli*. For the characteristics smoking habits (odds ratio (OR) 2.2; exact 95% CI 0.35–13.9; p = 0.4) and occupational exposure (OR 3.7; exact 95% CI 0.6–23.5; p = 0.16) the most elevated odds ratios were detected. However, no significant results were found for any of the odds ratios analyzed, which could be due to low numbers of samples included. The odds ratios should also be interpreted with caution because their confidence intervals include the ‘1’, which would display the case that no increased risk or protective effect is present for this parameter.

The analyses of the ‘high exposure’ group revealed a carriage of 10% (3/30) versus a carriage of 2.9% (2/69) in the ‘lower exposure’ group. For the job tasks included in the ‘high exposure group’, 11.1% of the workers involved in ‘evisceration’ (2/18) and 10% of workers of the team ‘hanging poultry in the slaughter process’ (1/10) carried ESBL-producing *E*. *coli* (Fig 3). Carriage of ESBL-producing *E*. *coli* detected in the collected samples indicated an association with occupational exposure in the working position (OR 3.7; exact 95% CI 0.6–23.5; p = 0.16).

## Discussion

The results of this preliminary study suggested that for slaughterhouse workers covering the work of earlier slaughtering steps (before cooling the carcasses), there might be an enhanced risk of carriage of ESBL-producing *E*. *coli* as colonizer in their intestines. The increased occupational exposure of workers in the process steps ‘hanging’ of poultry (10%) and ‘evisceration’ (11.1%) might be due to the uneven distribution of bacterial burden during the slaughter process. Previous publications have shown that the highest bacterial loads present in the first slaughtering process step, the ‘hanging of poultry’, decreases thereafter during the defeathering process, before increasing again after evisceration [23, 24]. After the cooling process, the bacterial loads decreased again to the allowed CFU/g [23]. Thus, the presence of ESBL-producing *Enterobacteriaceae* among poultry slaughterhouse workers covering working steps after cooling of the carcasses was lower (2.9%) than for the whole slaughterhouse. Therefore, it seems that there is no increased risk for steps handling poultry carcasses and meat, so that it might be a minor public health concern.

However to date, there is no national reference study in Germany analyzing the prevalence of ESBL-producing *Enterobacteriaceae* among the population. One study analyzing the intestinal colonization of Bavarian citizens detected a colonization of 6.3% [25], which is similar to the overall prevalence shown for the participants in the presented pilot study (5.1%). Although only a limited number of participants were involved in this pilot study and only few carriers were identified, which naturally influences the significance of the results, they can provide first hints of associations between occupational exposure and the colonization with ESBL-producing *E*. *coli*.

As ESBL-producing *E*. *coli* can be transmitted along the food chain, a higher prevalence in worker occupational exposed has been expected, concluding from the overall higher prevalence up to 44% of ESBL-producing *E*. *coli* detected in poultry husbandry [7, 26] and from poultry meat products [27, 28] compared to pig production of 15.2% [29–31]. However, the prevalence of ESBL-producing *E*. *coli* in participants working in poultry slaughterhouses was found to be comparable to that in participants working in pig slaughterhouses, with an overall prevalence of 4.8%. This raised the question whether the different slaughter technologies result in a similar low percentage of carriers of ESBL-producing *E*. *coli*, despite different prevalence in the animals slaughtered, as differences in slaughter techniques may result in less animal contact in poultry slaughter. The effect of hygienic measures is discussed controversially [32, 33]. Reduced occurrence of ESBL-producing *E*. *coli* in slaughterhouse workers could also be due to stricter hygiene measures in poultry than in pig slaughterhouses, as discussed by Schmithausen et al. (2015) [31]. However, further studies regarding the role of hygienic measures are necessary. The protective effect of the habit of smoking described in the study by Graveland et al. (2010) for MRSA cannot be shown for the colonization with ESBL-producing *E*. *coli* by the results of the present study [34].

In the current study, however, workers in the poultry slaughterhouse were found to have a similar tendency of ESBL carriage, depending on the exposure level, as Dohmen et al. (2017) already reported for workers in a pig slaughterhouse. In that study, the authors described an increased risk of intestinal colonization of slaughterhouse workers that belong to the ‘higher exposure group’, i.e., that have direct contact to live animals or the carcasses and organs at the beginning of the slaughter process.

The ESBL-genes detected in *E*. *coli* isolated from stool samples of the two employees, who had travelled in the last 12 months, were *bla*_SHV-12_ (isolate 3) and *bla*_CTX-M-15_ (isolate 2). In both cases, the acquisition of the ESBL-genes can be also due to different sources, as *bla*_CTX-M-15_ is found worldwide and *bla*_SHV-12_, represents an ESBL-gene with an association to poultry [8, 35]. Therefore, the increased chance of intestinal colonization due to traveling cannot be confirmed genotypically.

Nevertheless, three of five *E*. *coli* isolates carried the *bla*_*S*HV-12_ gene, which is frequently found in *E*. *coli* from poultry. The AmpC β-lactamase gene *bla*_CMY-2_, which is also frequently found in isolates from poultry, could not be detected in any of the isolates [27]. Overall, poultry cannot be regarded as the only source of the isolates, but it needs to be considered as one possible origin [8, 13].

Hospitalization and the use of antimicrobials, which have been described as possible risk factors in previous studies [36, 37], did not show an association with ESBL-carriage in the current study. While the numbers of non-ESBL carriers hospitalized (n = 16) and treated with antimicrobials (n = 14) was comparatively high, all ESBL-carriers reported a zero usage of antimicrobials and no hospitalization within the past 12 months. However, a study with an extended sample size could provide more evidence. For potential confounders, such as age and gender, neither literature on effects on ESBL status was found, nor were these identified within the present study.

Even though all the employees involved in the process of slaughter (n = 99) were included in the presented pilot study, the limited number of samples did not allow to determine significant associations within this pilot study. Therefore, larger studies are needed to determine the full influence of each characteristic. In this regard, it will be a major challenge of further research on the occupational exposure in poultry meat production to involve more participants in research studies, because due to the high mechanization of the process, far fewer people are employed in poultry slaughterhouses compared to pig slaughterhouses.

In addition, the risk, described for different working positions must be interpreted with caution, due to the fact, that the confidence interval of the presented odd ratios includes ‘1’. Therefore, the results of this pilot study for poultry are limited in their interpretation, as an increased risk cannot be extrapolated to poultry slaughterhouse workers in general. Further research including an extended sample size would also reduce this limitation.

Although the current study is based on a small number of participants, the findings represent the first data investigating an association between the occupational exposure of workers in a German poultry slaughterhouse and the intestinal colonization with ESBL-producing *Enterobacteriaceae*. Some working positions seem to represent an enhanced chance of intestinal colonization. Further research is needed to determine the full extent and effects of occupational exposure on the prevalence of ESBL-producing *E*. *coli* in slaughterhouse workers and to identify the effects of other risk and influencing factors.

## Supporting information

S1 Data (XLSX)
